# Identification of Regulatory Genes and Metabolic Processes Important for Alginate Biosynthesis in *Azotobacter vinelandii* by Screening of a Transposon Insertion Mutant Library

**DOI:** 10.3389/fbioe.2019.00475

**Published:** 2020-01-17

**Authors:** Mali Mærk, Øyvind M. Jakobsen, Håvard Sletta, Geir Klinkenberg, Anne Tøndervik, Trond E. Ellingsen, Svein Valla, Helga Ertesvåg

**Affiliations:** ^1^Department of Biotechnology and Food Science, Norwegian University of Science and Technology, Trondheim, Norway; ^2^SINTEF Industry, Trondheim, Norway

**Keywords:** alginate, *Azotobacter vinelandii*, *amrZ*, *fruA*, *algB*, medium supplements

## Abstract

*Azotobacter vinelandii* produces the biopolymer alginate, which has a wide range of industrial and pharmaceutical applications. A random transposon insertion mutant library was constructed from *A. vinelandii* ATCC12518Tc in order to identify genes and pathways affecting alginate biosynthesis, and about 4,000 mutant strains were screened for altered alginate production. One mutant, containing a *mucA* disruption, displayed an elevated alginate production level, and several mutants with decreased or abolished alginate production were identified. The regulatory proteins AlgW and AmrZ seem to be required for alginate production in *A. vinelandii*, similarly to *Pseudomonas aeruginosa*. An *algB* mutation did however not affect alginate yield in *A. vinelandii* although its *P. aeruginosa* homolog is needed for full alginate production. Inactivation of the fructose phosphoenolpyruvate phosphotransferase system protein FruA resulted in a mutant that did not produce alginate when cultivated in media containing various carbon sources, indicating that this system could have a role in regulation of alginate biosynthesis. Furthermore, impaired or abolished alginate production was observed for strains with disruptions of genes involved in peptidoglycan biosynthesis/recycling and biosynthesis of purines, isoprenoids, TCA cycle intermediates, and various vitamins, suggesting that sufficient access to some of these compounds is important for alginate production. This hypothesis was verified by showing that addition of thiamine, succinate or a mixture of lysine, methionine and diaminopimelate increases alginate yield in the non-mutagenized strain. These results might be used in development of optimized alginate production media or in genetic engineering of *A. vinelandii* strains for alginate bioproduction.

## Introduction

Alginate is the collective term for a family of linear polysaccharides consisting of varying amounts of β-D-mannuronic acid (M) and α-L-guluronic acid (G) (Haug et al., 1966). In nature alginates are produced by brown seaweeds and by several bacteria in the genera *Pseudomonas* and *Azotobacter*, among them the soil bacterium *A. vinelandii* (Gorin and Spencer, 1966). Bacterial alginate is produced as an exopolysaccharide, and the biosynthetic apparatus is similar in both genera (Ertesvåg, 2015). However, *A. vinelandii* produces alginate constitutively, while biosynthesis is activated only under certain environmental conditions in *Pseudomonas* spp.

Alginates are commercially important biopolymers with a wide range of industrial and technological applications (Skjåk-Bræk et al., 2015). All commercial alginate production is currently based on extraction from brown algae, which yields complex mixtures of alginates with regard to both chain composition and molecular weight (Andersen et al., 2012). The bulk alginates used in for example food and cosmetic industry can be acquired at prices as low as 5 USD per kilogram, while pharmaceutical or medical grade alginates with defined molecular weights and M/G profiles, and thus more defined material properties, cost about 100 USD per gram (NovaMatrix web catalog prices, September 2019). New pharmaceutical and medical applications are being developed, and this increases the demand for high-end alginates. Because engineered bacteria have the potential for *in vivo* production of homogeneous alginates with specialized compositions (Remminghorst and Rehm, 2006), there is considerable interest in microbial bioproduction of these polymers. Furthermore, as most current applications rely on the gelling properties of G-blocks (stretches of consecutive G residues) (Andersen et al., 2012), *A. vinelandii* is an attractive candidate for strain engineering due to its innate ability to introduce G-blocks in the alginate chains (Ertesvåg et al., 1995; Svanem et al., 1999). No strains producing G-block alginates have been identified among the *Pseudomonas* species studied so far.

In order to optimize bacterial production processes, it is important to understand how alginate biosynthesis is regulated as well as elucidate which other metabolic aspects have an influence on production levels. The precursor for the alginate monomers is fructose 6-phosphate, so alginate biosynthesis is closely connected to the central carbohydrate metabolism of the cells (Maleki et al., 2015, 2017; Ertesvåg et al., 2017). Alginate production is furthermore an energy demanding process and is tightly controlled by a complex network of regulators which also influence other cellular processes (Urtuvia et al., 2017). The state of several cellular processes can therefore be expected to affect the biosynthesis of alginate. So far, the regulatory network controlling alginate biosynthesis has mainly been studied in *P. aeruginosa*. Regulatory differences between *P. aeruginosa* and *A. vinelandii* have already been observed (Núñez et al., 1999; López-Pliego et al., 2018), demonstrating a need for further investigations of these mechanisms in the latter organism. In addition, previous investigations of *A. vinelandii* have mainly been directed toward gene disruptions or environmental factors leading to an increase in alginate biosynthesis (Núñez et al., 2013; Ahumada-Manuel et al., 2017; Quiroz-Rocha et al., 2017). Identification of genes and processes which influence alginate biosynthesis negatively is however equally important in order to achieve a better understanding of limiting factors, which is a central issue when industrial production purposes are considered.

The aim of this study was to investigate factors affecting alginate biosynthesis in *A. vinelandii* by screening of a transposon insertion library of strain ATCC12518Tc, a tetracycline-resistant derivative of ATCC12518. The genome of *A. vinelandii* DJ, also a derivative of ATCC12518, has been sequenced (Setubal et al., 2009), thus greatly simplifying identification of the affected genes in transposon insertion mutants with interesting phenotypes. We have previously screened a *Pseudomonas fluorescens* transposon library (Ertesvåg et al., 2017), and an additional aim of the current study was to compare the results from the two bacteria in order to detect similarities and differences between the two genera.

## Materials and Methods

### General Cultivation of Bacteria

The bacterial strains and plasmids used in this work are described in Table 1. *Escherichia coli* strains were routinely grown in LB broth (10 g/l tryptone, 5 g/l yeast extract, 5 g/l NaCl) or on LB agar at 37°C. *A. vinelandii* strains were routinely grown in liquid Burk's medium (BM) or RA1 medium (Gimmestad et al., 2006) supplemented with 3.0 ml/l TMS1 (Wentzel et al., 2012) or on BM or RA1 agar at 30°C. The media contained 20 g/l of the relevant carbon source unless stated otherwise. Biotin (1.6 μM), lysine (0.5 mM), methionine (0.6 mM), diaminopimelate (0.2 mM), adenine (0.8 mM), thiamine (2 μM), pyridoxine (5 μM), pyridoxal (5 μM), or succinate (30 mM) were added in some experiments. Antibiotics were present in the following concentrations when used in cultivations: ampicillin 200 μg/ml, tetracycline 15 μg/ml, spectinomycin 20 μg/ml, apramycin 25 μg/ml (*A. vinelandii*) or 50 μg/ml (*E. coli*), kanamycin 2 μg/ml (*A. vinelandii*) or 50 μg/ml (*E. coli*).

**Table 1:** Bacterial strains and plasmids used in this work.

**Strain or plasmid**	**Description**	**References**
**STRAINS**		
*Escherichia coli*		
S17.1	RP4 2-Tc::Mu-Km::*Tn7pro, res, mod*	Simon et al., 1983
S17.1 (λpir)	λ*pir* (for replication of oriR6K-plasmids) *recA, thi pro hsdR-M^+^* RP4 2-Tc::Mu-Km::*Tn7*Tp^r^ Sm^r^	de Lorenzo et al., 1993
*Azotobacter vinelandii*		
ATCC12518	Alginate producing wild type strain.	ATCC
ATCC12518Tc	Derivative of ATCC12518 where the *tetAR* genes from pLit28Tc are inserted in one of the four homologous transposase genes *Avin09530, Avin31560, Avin36480*, and *Avin49630*. (Exact location not determined because these homologs differ in only 1 bp.) Tc^r^	This work
**PLASMIDS**		
pCAM140	Delivery vector of mini-Tn5. Ap^r^, Sp^r^	Wilson et al., 1995
pLit28Tc	ColE1. Tc^r^, Ap^r^	Bakkevig et al., 2005
pKD21	Tn5-based mini-transposon vector encoding luciferase controlled by a mutant Pm-promoter. Km^r^.	Bakkevig et al., 2005
pIB11	RK2 based expression vector using the inducible *Pm*-XylS promoter system. Km^r^.	Bakke et al., 2009
pHE206	Derivative of pLit28Tc containing a 1.0 kb KpnI-HindIII restricted PCR fragment encoding the 3′ end of the putative transposase gene *Avin31560* downstream of *tetR*. Tc^r^, Ap^r^	This work
pHE208	Derivative of pHE206 containing a 1.5 kb SpeI-XhoI restricted PCR fragment encoding the 5′ end of *Avin31560* and the upstream *ispB*-like gene upstream of *tetA*, resulting in a gene replacement vector containing *Avin31560* disrupted by insertion of the *tetA*-*tetR* genes. Tc^r^, Ap^r^	This work
pMH13	Derivative of pKD21 containing wild type Pm-promoter. *neo* replaced by apramycin resistance gene. Am^r^	This work
pHE319	Derivative of pMH13 in which the luciferase gene was replaced by a 1.9 kb PCR-fragment encoding *dxs-1*. Am^r^	This work
pHE536	Derivative of pIB11 where a 1.2 kb PCR fragment encoding *algW* replaced *bla*. Km^r^.	This work
pHE537	Derivative of pIB11 where a 0.4 kb PCR fragment encoding *amrZ* replaced *bla*. Km^r^.	This work
pHE542	Derivative of pIB11 where a 2.0 kb PCR fragment encoding *fruA* replaced *bla*. Km^r^.	This work

### Standard Procedures

Plasmid isolations, enzymatic DNA manipulations and agarose gel electrophoresis were performed according to Sambrook and Russell (2001). Transformation and conjugation was performed as described earlier (Gimmestad et al., 2009). Pwo SuperYield DNA Polymerase (Roche Diagnostics) or Q5 polymerase (New England Laboratories) was used to amplify DNA from *A. vinelandii*. The QIAquick Gel Extraction Kit and QIAquick PCR Purification Kit (QIAGEN) were used for DNA purifications from gel electrophoresis and enzymatic reactions, respectively. Chromosomal DNA was isolated from *A. vinelandii* strains using the PurElute™ Bacterial Genomic Kit (EdgeBio). Cells were washed with 0.9% NaCl, 10 mM EDTA (pH 8.0) prior to DNA isolation in order to remove extracellular alginate. DNA sequencing was performed using the BigDye® Terminator v1.1 Cycle Sequencing Kit (Applied Biosystems). Transposon insertion sites were identified using primers that are complementary to the ends of the inserted fragment, and genomic DNA isolated from each mutant as the template, thus allowing direct sequencing of the genomic regions flanking the insertion. Primer sequences for PCR and sequencing are available upon request.

### Construction of a Transposon Insertion Mutant Library

The mini-Tn5 transposon delivery vector pCAM140 which encodes a spectinomycin resistance gene flanked by transcriptional terminators to avoid read-through from the transposon (Wilson et al., 1995) was introduced to *A. vinelandii* ATCC12518Tc by conjugation. Mating was performed at 30°C on LB agar and transconjugants were selected on RA1 agar containing tetracycline and spectinomycin. Selection plates also contained alginate lyase AlgL (6.5 mU/cm^2^) (Ertesvåg et al., 1998); the enzyme was applied to the plates before spreading the cells in order to reduce the colony mucoidicity of alginate-producing transconjugants. Transconjugant colonies were picked using a Genetix Q-Pix2 robotic colony picker and transferred to 96-well microtiter plates containing 110 μl liquid 0.5x RA1 medium (CaCl_2_·2H_2_O and MOPS concentrations as for 1x medium, and 10 g/l fructose) with tetracycline and spectinomycin. *A. vinelandii* insertion mutants are known to sometimes contain copies of both mutant and wild-type alleles, so to eliminate wild-type chromosome copies the transconjugants were grown by repeated transfers in selective media. All liquid and microtiter plate handling were performed by a Beckman Coulter Core robotic equipped with a Beckma Coulter NXP liquid handling unit.

### Alginate Analyses

Culture samples (diluted in 0.2 M NaCl when necessary to reduce viscosity) were centrifuged to remove bacterial cells, and the alginates in the cell-free supernatants were deacetylated by mild alkaline treatment as described previously (Ertesvåg and Skjåk-Bræk, 1999). The alginate content in the deacetylated samples was determined enzymatically as described earlier (Østgaard, 1992; Maleki et al., 2015). Samples for alginate quantification were collected from transconjugants cultivated in 96-well microtiter plates with 110 μl selective 0.5x RA1 medium in each well. Mutants displaying a potential increase or decrease in alginate production were verified by assessing alginate production in triplicate cultures in 96-deepwell plates with 600 μl selective 0.2x RA1 medium in each well, or in 250 ml shake flask with 30 ml 0.5x RA1 medium. In all experiments, *A. vinelandii* ATCC12518Tc was included as a reference. The reference strain displayed similar alginate production and growth characteristics in deepwell plates and in shake flasks. The final alginate production and cell densities reported in this study are based on measurements of extracellular alginate concentration (described above) and OD_660_ respectively, sampled in stationary phase.

For qualitative analyses of complemented strains, 1 ml culture was centrifuged, NaCl was added to the supernatant (0.1 M) and the alginate was precipitated with an equal amount of isopropanol. Only alginate-producing strains produce a pellet in this assay. The mutant strain, or mutant strain with an empty vector (pIB11), was always used as a control.

### Fermentations

Fermentations of *A. vinelandii* strains were performed as described by Steigedal et al. (2008) except that the inoculum was cultivated in two steps in 0.5x RA1 medium in shake flasks. The first culture was cultivated until visible growth, followed by inoculating 3% into fresh medium for the second stage inoculum, which was then cultivated over night before being transferred, 3%, to 3 l Applikon fermentors with 1 l of 0.5x PM1 containing the following ingredients per liter: fructose (50 g), peptone (4.75 g), MgSO_4_·7H_2_O (0.3 g), KH_2_PO_4_ (65 mg), K_2_HPO_4_ (16 mg), NaCl (0.2 g), CaCl_2_·2H_2_O (0.29 g), FeSO_4_·7H_2_O (20 mg), and Clerol FBA622 (antifoam, 0.5 g). The fermentations were performed at 30°C. pH was adjusted to 7.2 from start and controlled at this pH by addition of NaOH. The dissolved oxygen was controlled to 10% of saturation by automatic control of the stirrer speed. Aeration rate in the culture medium in fermentors was initially 0.25 vvm (l_gas_/l_liquid_ per minute) and was increased up to 1.0 vvm when required for maintaining dissolved oxygen without exceeding the maximum stirrer speed of the fermentor (2,000 rpm).

## Results

### Construction and Screening of the Transposon Insertion Mutant Library

In order to obtain an unbiased transposon insertion library allowing for auxotrophic mutants, the strains have to be maintained on a rich medium. This required insertion of a resistance marker into the *A. vinelandii* ATCC12518 strain, to allow for counter selection of *E. coli* after conjugation. Using the gene replacement vector pHE208, we therefore constructed a tetracycline resistant derivative of ATCC12518, designated ATCC12518Tc, in which a putative transposase gene is disrupted by insertion of the *tetA-tetR* genes (Table 1). Strain ATCC12518Tc produced 8.3 (±0.9) g/l alginate in batch fermentations, which is about 70% of that observed for the wild type. The reason for this reduction is not known. The production level is however still sufficient to allow for identification of mutants with lowered alginate production, and the decrease could potentially be beneficial for identification of mutants with increased production. A transposon insertion library of ~4,000 transconjugants was made from *A. vinelandii* ATCC12518Tc as described in the Materials and Methods section.

The mutant strains from the library were cultivated in 96-well microtiter plates and screened with regard to alginate production levels compared to the reference strain ATCC12518Tc (see Materials and Methods section). Mutants that failed to grow (~1,000 mutants) were disregarded. Among the viable mutants, the screen identified 56 mutants with potentially increased alginate production (>125% alginate concentration relative to the reference strain) and 241 mutants with potentially lowered alginate production (<50% relative to the reference).

### Verification of Mutants With Altered Alginate Production Levels

Selected mutants with potentially altered alginate production were recultivated in triplicates in deepwell plates and/or shake flasks (see Materials and Methods section) for verification of the initial screening results. A total of 17 mutants with potentially increased alginate production and 109 mutants with potentially lowered alginate production were chosen for verification experiments. Using the same criteria as above, an increase or decrease in alginate production relative to the reference strain was affirmed for 2 and 68 of these candidates, respectively. The verified mutants showing increased or decreased alginate production are hereafter referred to as up-mutants and down-mutants, respectively, and their alginate production and growth levels relative to the reference strain is given in Table 2. Several of the mutant strains were observed to aggregate in liquid culture. Thus, growth measured as OD_660_ could not always be considered a reliable measure and is therefore not discussed in more detail. It should however be noted that for one of the up-mutants, with a transposon insertion in *pgm-2*, the apparent increase in alginate yield is most likely caused by an increased cell density in the cultures (Table 2).

**Table 2:** *A. vinelandii* transposon insertion mutants displaying altered alginate production levels.

**Metabolic category**	**Strain**	**GeneID for inactivated gene**	**Gene name**	**Gene product**	**Relative growth**	**Relative alginate production**
Reference strain	ATCC12518Tc				1.0	1.0
**Mutants with lowered alginate production (deepwell plate cultivations)**						
Aromatic compounds	33F08	Avin08040		Aromatic acid decarboxylase	0.7	0.1
Biosynthesis of cofactors	27H10	Avin05990	*bioF*	8-amino-7-oxononanoate synthase	0.8	0.1
	33A03	Avin07870	*dxs-1*	1-deoxy-D-xylulose 5-phosphate synthase	0.6	0.0
Cell envelope	03E03	Avin05370		Lipopolysaccharide biosynthesis protein	0.6	0.1
	03G10	Avin15980		Glycosyl transferase	0.7	0.4
	30F06	Avin26720		OmpA/MotB domain protein	0.7	0.0
Cellular processes	14B06	Avin45390	*hslU*	Heat shock protein	0.7	0.3
	20A04	Avin12950	*algW*	Htr-like protease	0.7	0.0
	22B07	Avin27920	*flhB*	Flagellar biosynthetic protein	0.7	0.1
	25H12	Avin35740		Conjugation protein	0.9	0.3
	37D04	Avin41270		Peptidase M48	0.7	0.0
Central intermediary metabolism	13E05	Avin31330		Alkanesulfonate monooxygenase	0.7	0.3
	29D11	Avin18740		Acetyl CoA hydrolase/transferase	0.6	0.1
	31H08	Avin25610		TauD/TfdA dioxygenase family protein	0.7	0.0
	26B04	Avin08860 and/or Avin08880		Phenol hydroxylase subunit (DmpK) and/or sigma54-dependent activator protein	0.7	0.2
DNA metabolism	15B03	Avin20500		Phage integrase	1.0	0.1
	27D11	Avin52330		Type III restriction enzyme Res subunit	0.7	0.2
Energy metabolism	08B10	Avin21890		Monooxygenase	0.9	0.3
	08E05	Avin12210	*fruA*	Fructose PTS IIBC	0.6	0.0
	20A06	Avin29770	*sucA*	2-oxoglutarate dehydrogenase E1 component	0.8	0.0
	22H06	Avin28560	*nuoN*	Proton-translocating NADH-quinone oxidoreductase	0.6	0.1
	36F10	Avin26020	*gcvP2*	Glycine dehydrogenase	0.4	0.2
Extracellular polysaccharides	10B06	Avin10970	*algD*	GDP-mannose 6-dehydrogenase	0.7	0.1
	12B08	Avin10970	*algD*	GDP-mannose 6-dehydrogenase	0.5	0.1
	25G08	Avin10970	*algD*	GDP-mannose 6-dehydrogenase	0.8	0.1
	43G02	Avin10970	*algD*	GDP-mannose 6-dehydrogenase	0.8	0.1
	10E01	Avin10860	*algA*	Mannose 1-phosphate guanylyltransferase/mannose 6-phosphate isomerase	0.8	0.2
	12G04	Avin10940	*algK*	Alginate biosynthesis protein	0.6	0.0
	19H12	Avin10930	*algJ*	Alginate biosynthesis protein	0.7	0.1
	29H08	Avin10930	*algJ*	Alginate biosynthesis protein	0.7	0.1
	21F06	Avin10890	*algI*	Alginate O-acetyl transferase	0.9	0.1
	35G10	Avin10890	*algI*	Alginate O-acetyl transferase	0.7	0.1
Hypothetical	13F07	Avin31830		Hypothetical protein	0.8	0.3
	15E07	Avin09340		Hypothetical protein	0.5	0.1
	15H03	Avin41170		Hypothetical protein	0.9	0.1
	24A04	Avin36260		Hypothetical protein	0.6	0.1
	24F09	Avin28850		Hypothetical protein	0.6	0.1
	26C02	Avin11200		Membrane protein	0.7	0.2
	26D10	Avin39360		Hypothetical protein	0.7	0.1
	31G08	Avin33410		Hypothetical protein	0.8	0.1
	32E09	Avin34870		Hypothetical protein	0.9	0.1
	36A03	Avin16680/−20990		Hypothetical protein	1.0	0.1
	39D11	Avin43510		Hypothetical protein	0.7	0.1
Lipids	07B03	Avin29550	*arsB*	Type III PKS	0.6	0.0
	10E09	Avin13550		Enoyl-CoA hydratase/isomerase	0.9	0.1
Other	16H06	Avin21160		Enterobactin domain protein	0.7	0.1
	29F10	Avin05960		Aminoglycoside phosphotransferase	0.8	0.3
	31H01	Avin32770		Metallophosphoesterase	0.6	0.2
	32B04	Avin25650		NRPS: amino acid adenylation	0.5	0.1
Purines and pyrimidines	22G07	Avin39660	*purL*	Phosphoribosylformyl-glycinamidine synthase	0.8	0.0
	32A11	Avin02510		Thymidylate kinase	0.7	0.1
Regulatory functions	09D06	Avin34410	*amrZ*	Alginate and motility regulator Z DNA binding protein	0.8	0.0
	22F02	Avin34410	*amrZ*	Alginate and motility regulator Z DNA binding protein	0.8	0.0
	39H08	Avin34410	*amrZ*	Alginate and motility regulator Z DNA binding protein	0.7	0.2
	21A12	Avin18010	*dctB*	C4-dicarboxylate transport sensory histidine protein kinase	0.6	0.1
	21C03	Avin13880		Transcriptional regulatory protein	1.0	0.1
	31F07	Avin10390		LysR family regulatory protein	0.8	0.1
	39C09	Avin38020		Transcriptional regulator	0.7	0.1
	42G12	Avin32720		Response regulator	0.9	0.4
	49G12	Avin18640		Sensory histidine protein kinase	0.7	0.1
Translation	43C11	Avin23730		Modification methylase	0.7	0.1
Transport and binding proteins	01A04	Avin14160		ABC transporter component	0.6	0.1
	10D12	Avin12330		TonB-dependent siderophore receptor	0.6	0.1
	27D02	Avin40960		ABC transporter component	0.5	0.0
	28A08	Avin14340		Acriflavin resistance protein	0.9	0.1
	28D07	Avin47130		TonB-dependent receptor	0.7	0.2
	38D10	Avin19760		Phosphonate ABC transporter	0.5	0.1
Transposon	40A06	Avin09580/- 10840/- 15010/- 23940/- 33330/- 36160/- 49850		Transposase	0.6	0.1
**Mutants with increased alginate production (shake flask cultivations)**						
Energy metabolism	39G08	Avin27440	*pgm-2*	2,3-bisphosphoglycerate-independent phosphoglycerate mutase	1.4	1.3
Transcription	36C11	Avin13700	*mucA*	Sigma factor AlgU negative regulatory protein	0.7	1.7

*Alginate production and cell growth (OD_660_) as observed in deepwell plate or shake flask cultivations, given as fractions normalized relative to that of the reference strain ATCC12518Tc. (Defined as 1.0 for the reference strain, which had an average growth of OD660 and an average alginate production of 7 g/l). The results shown are average values based on triplicate cultivations. The inactivated gene, identified by insertion point sequencing, is given for each mutant. Sequencing also showed that none of the mutants with insertions in the same gene have the same insertion point, and thus result from independent insertion events. Known or putative gene names, gene products and metabolic categories were retrieved from the A. vinelandii DJ genome database (Setubal et al., 2009). See text for discussion of specific mutants*.

### Validation of the Verification Process by Batch Fermentation Trials

To further confirm the alginate phenotypes observed in the verification experiments, fermentations were performed for 12 of the down-mutants and one up-mutant (36C11) in order to obtain growth and alginate production data under highly controllable conditions. Very low or no alginate production (<10% relative to the reference strain) was confirmed for 10 of the down-mutants (27H10, 33A03, 20A04, 22B07, 08E05, 20A06, 36F10, 22G07, 39H08, and 21C03), while the remaining two were shown to produce 67% (03E03) and 35% alginate (45G12) compared to the reference strain. Batch fermentation of the up-mutant 36C11 confirmed a significant increase in alginate production; 180% relative to ATCC12518Tc. The general agreement between the fermentation results and the alginate production levels observed in deepwell plates indicate that verification experiments with triplicate deepwell plate cultivations can be regarded as a reliable method for evaluating large numbers of *A. vinelandii* strains with regard to alginate production.

### Identification of Disrupted Genes in Mutants With Altered Alginate Production

The transposon insertion points were identified in the genomes of the verified up- and down-mutants (Table 2) by determining the chromosomal DNA sequences flanking the transposons and performing BLAST (Altschul et al., 1997) searches against the *A. vinelandii* DJ genome (Setubal et al., 2009). The affected genes include genes involved in metabolism, transport, translation and gene regulation (Table 2). For 11 of the mutants in Table 2, the affected genes were annotated as hypothetical. Ten of the mutants have insertions in structural genes directly involved in alginate biosynthesis (*algD, algK, algJ, algI*, and *algA*) (Rehm et al., 1996), and show the expected *alg*^−^ phenotype. For further investigations of selected mutants, we chose to focus on some of the genes with known or putative regulatory functions. Moreover, in an earlier study on the alginate-producing mutant of *P. fluorescens*, it was reported that inactivation of many genes in the central metabolism resulted in lower alginate production. We therefore wanted to investigate whether this appeared to be the case for *A. vinelandii* as well.

### Transposon Insertions in Regulatory Genes Presumed to Be Involved in Alginate Biosynthesis

The regulation of alginate biosynthesis has mostly been studied in *P. aeruginosa* (reviewed in Hay et al., 2014; Urtuvia et al., 2017). The phenotypes of *A. vinelandii* mutants found to have insertions in such genes are therefore discussed taking *P. aeruginosa* strains with disruptions in homologous genes into consideration. One mutant was found to have the transposon inserted in the known *A. vinelandii* alginate regulatory gene *mucA*, and five mutants had insertions in homologs of alginate regulatory genes known from *P. aeruginosa*: *algW, amrZ* (three independent mutants), and *algB*. These mutants are described below. The gene context of the genes discussed below are shown in Supplementary Figure S1. In addition, several other genes putatively involved in transcription regulation were identified in the screen (Table 2), but since their targets are unknown, they were not studied further.

MucA is an anti-sigma factor that represses sigma factor AlgU, which is needed for expression of alginate biosynthetic gene *algD* (and possibly other alginate biosynthetic genes). *A. vinelandii mucA* mutants have previously been shown to display increased alginate production (Martínez-Salazar et al., 1996; Núñez et al., 2000). As could be expected, this is also the case for the *mucA*::TnCAM140 mutant identified in this work.

The protease AlgW has been shown to be required for activation of alginate biosynthesis in *P. aeruginosa*, by degrading MucA in a MucE-dependent manner (Wood et al., 2006; Qiu et al., 2007; Cezairliyan and Sauer, 2009). The *algW*::TnCAM140 mutant identified in this work does not produce alginate, which indicates that the *A. vinelandii* AlgW homolog has a function similar to the *P. aeruginosa* protease even though there is no *mucE* homolog in *A. vinelandii* (Setubal et al., 2009). However, when complemented with *algW* encoded on plasmid pHE536, the alginate production was not restored.

An *algB*::TnCAM140 mutant was identified among the mutants with lowered alginate production in the initial screen, but further evaluations showed that the production level of this mutant is actually comparable to that of the reference strain. The two-component response regulator AlgB is required for alginate production in *P. aeruginosa* (Goldberg and Ohman, 1987; Goldberg and Dahnke, 1992; Wozniak and Ohman, 1994) where it positively regulates expression of biosynthetic genes by direct binding to P*algD* (Leech et al., 2008). Our results thus indicate that the role of the AlgB homolog in *A. vinelandii* differs from that of *P. aeruginosa* AlgB. PCR on chromosomal DNA from the transposon mutant confirmed the absence of wild type copies of the *algB* gene. Thus, it appears that AlgB is not required for alginate production in *A. vinelandii*.

AmrZ (AlgZ) is a DNA binding protein required for transcription from P*algD* in *P. aeruginosa* (Baynham et al., 1999), and has also been shown to negatively regulate motility in both *P. aeruginosa* and *P. fluorescens* via FleQ (Baynham et al., 2006; Tart et al., 2006; Martínez-Granero et al., 2012). Expression of *P. aeruginosa amrZ* is controlled by the sigma factor AlgU (AlgT) (Wozniak et al., 2003). AlgU negatively regulates motility in *A. vinelandii* as well, but via CydR and FlhDC instead of AmrZ and FleQ (León and Espín, 2008). An *amrZ* mutant has not previously been described in *Azotobacter*. The alginate production in our *amrZ*::TnCAM140 mutant was restored by complementation using plasmid pHE537, which encodes *amrZ*. This confirms that AmrZ is necessary for alginate production in *A. vinelandii*, like it is in *P. aeruginosa*.

### A *fruA* Transposon Insertion Abolishes Alginate Production in *A. vinelandii*

Alginate production was absent in a mutant with the transposon insertion in an *E. coli fruA* homolog. Complementation with plasmid pHE542 encoding *fruA* restored the strain's ability to produce alginate (data not shown), demonstrating that the observed phenotype was indeed caused by the *fruA* disruption. As FruA is a part of the fructose phosphoenolpyruvate phosphotransferase system (PTS) (Prior and Kornberg, 1988), the lack of alginate synthesis could simply be caused by limited carbon uptake, since fructose was used as the carbon source in cultivations. The *fruA*::TnCAM140 mutant was therefore evaluated in media containing other carbon sources, but still did not produce alginate when fructose was replaced with glucose, glycerol or sucrose (data not shown).

### Mutations in Genes Encoding Proteins Involved in Biosynthesis of Vitamins, Cofactors and Biosynthetic Precursors Affect Alginate Production Levels

**Figure 1:**
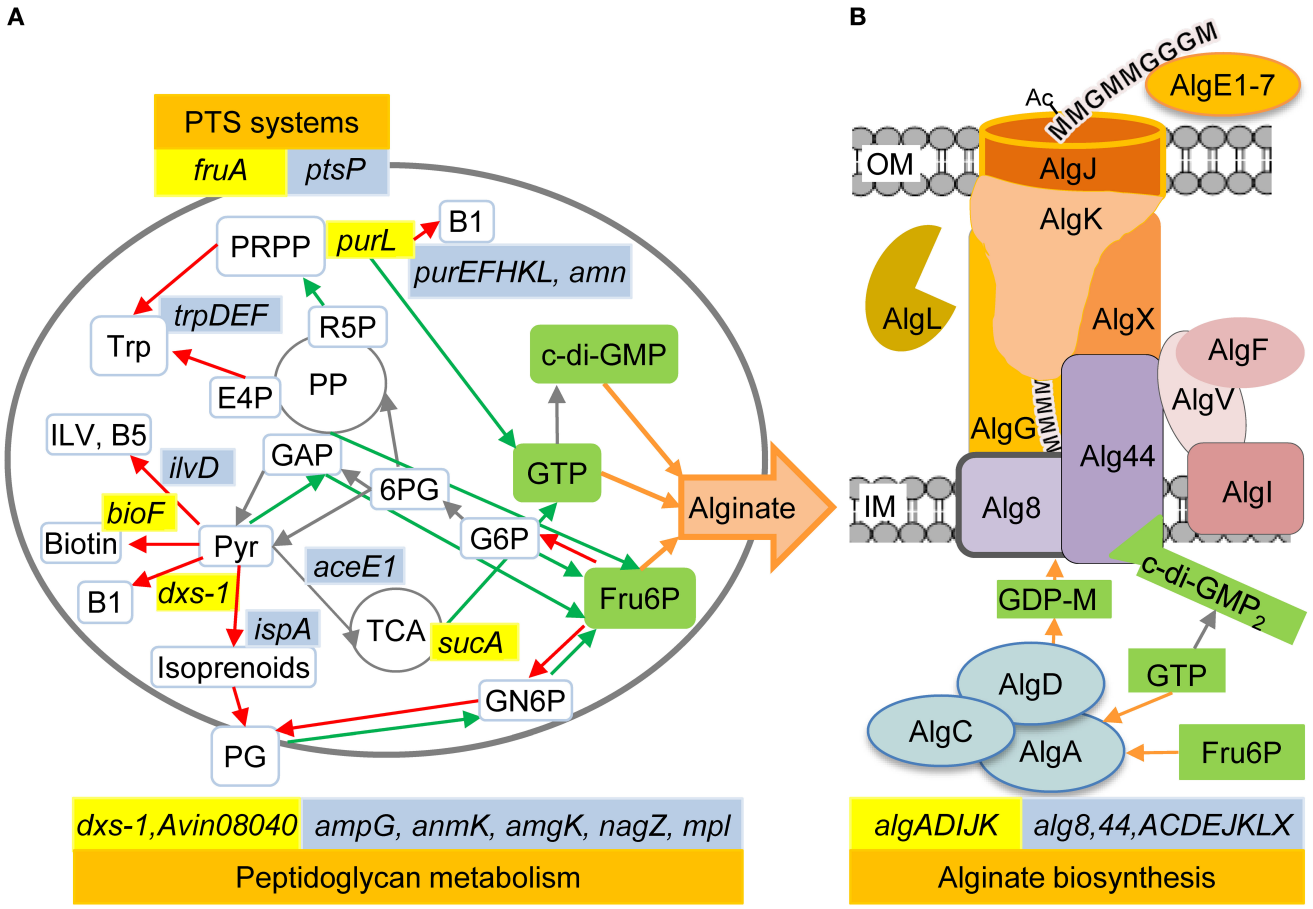
The relationship between alginate biosynthesis and the cellular metabolism in *A. vinelandii* and *P. fluorescens*. **(A)** A simplified model of the cell's metabolism highlighting the processes identified in the present study as being important for full alginate biosynthesis levels. **(B)** The proteins and metabolites directly needed for alginate biosynthesis (adapted from Ertesvåg et al., 2017). *A. vinelandii* genes discussed in the current paper are highlighted in yellow, while the previously identified *P. fluorescens* genes are highlighted in blue. In *P. fluorescens*, the AlgJ homolog is named AlgE, while the AlgV homolog is named AlgJ. Red arrows indicate pathways competing with accumulation of the three metabolites Fru6P, GTP, and c-di-GMP, which are essential for alginate biosynthesis, while green arrows indicate pathways that would increase the synthesis of one of these three metabolites. Each arrow may represent several enzymatic steps. OM, Outer membrane; IM, Inner membrane; 6PG, 6-Phosphogluconate; Ac, Acetyl; B1, Thiamine; B5, Pantothenate; E4P, Erythrose 4-phosphate; G, Guluronic acid residue; GN6P, Glucosamine 6-phosphate; G6P, Glucose 6-phosphate; ILV, Isoleucine Leucine Valine; M, Mannuronic acid residue; PG, Peptidoglycan; PP, Pentose-phosphate cycle; PRPP, Phosphoribosyl pyrophosphate; Pyr, Pyruvate; R5P, Ribose 5-phosphate; TCA, Tricarboxylic acid cycle; Trp, Tryptophan.

Transposon insertions in genes involved in isoprenoid, purine and thiamine biosynthesis resulted in decreased alginate production in *A. vinelandii*, and the same has previously been observed for *P. fluorescens* (Figure 1). Several other *A. vinelandii* mutants shown to produce very little or no alginate have transposon insertions in genes involved in the biosynthesis of cofactors (vitamins) or central metabolic precursors (Table 2). Selected mutants in these categories are described below.

1-deoxy-D-xylulose-5-phosphate synthase (Dxs) synthesizes the common precursor for thiamine, pyridoxine, and isoprenoid biosynthesis (Sprenger et al., 1997). An *A. vinelandii dxs-1* mutant identified in our screen was found to produce no or very low amounts of alginate. *A. vinelandii* has two nearly identical *dxs* genes, but the phenotype of the *dxs-1* mutant indicates that the expression of *dxs-2* alone is not sufficient for alginate production. Production was restored by cloning wt *dxs-1* on a transposon and transferring it back to the *dxs-1*::TnCAM140 mutant (data not shown), thus confirming that *dxs-1* is required for alginate production in *A. vinelandii*. Cultivating the *dxs-1*::TnCAM140 mutant with pyridoxine, pyridoxal or thiamine added to the growth medium did however not restore alginate production (data not shown).

The phosphoribosylformylglycinamidine synthase PurL is involved in *de novo* biosynthesis of purines (Sampei and Mizobuchi, 1989). A *purL* mutation abolished alginate production in both *A. vinelandii* (this study) and *P. fluorescens* (Ertesvåg et al., 2017). For *P. fluorescens* this was also observed for several additional mutants where genes involved in either *de novo* or salvage pathways for purine synthesis were disrupted (Figure 1). Some of these *P. fluorescens* mutants were complemented, and complementation was shown to restore alginate biosynthesis (Ertesvåg et al., 2017).

PurL is also involved in biosynthesis of thiamine, as thiamine is derived from the purine biosynthesis intermediate 5-amino-1-(5-phospho-D-ribosyl)imidazole (AIR) (Begley et al., 1999). Thiamine is an essential cofactor for a variety of enzymes, several of which play important roles in carbohydrate metabolism. Due to the central roles of purines and thiamine, *A. vinelandii* cells that do not synthesize these compounds would be expected to be unable to grow in the media used in this study. *A. vinelandii* insertion mutants are however known to sometimes contain copies of both mutant and wild-type alleles. This was shown to be the case for the *purL*::TnCAM140 mutant, which explains how the strain was still able to grow normally.

Likewise, a low-alginate-producing *bioF*::TnCAM140 mutant was shown to retain wild-type alleles. A genetically pure *bioF* mutant would not be expected to grow in unsupplemented media, as BioF is an 8-amino-7-oxononanoate synthase involved in the synthesis of biotin, a cofactor required for cellular growth (Marquet et al., 2001).

Our results also revealed that alginate production was abolished in an *A. vinelandii sucA*::TnCAM140 mutant. SucA is part of the 2-oxoglutarate dehydrogenase complex, which catalyzes the irreversible conversion of 2-oxoglutarate to succinyl-CoA in the tricarboxylic acid (TCA) cycle. In addition to being an intermediate in the TCA cycle, succinyl-CoA is a precursor for several important biomolecules. Inactivation of *sucA* would be expected to reduce growth rate (Yu et al., 2006), but PCR showed that the *sucA* mutant, like the *purL* and *bioF* mutants, has retained both disrupted and wild-type alleles, which can explain why growth was unimpaired (Table 2).

### Alginate Production Is Limited by Access to Key Metabolites in *A. vinelandii*

As mentioned earlier, *A. vinelandii* has multiple copies of its chromosome and will use this to retain wild type copies of genes necessary to maintain an adequate growth rate even if the inactivated version is selected for by an antibiotic. As described above, the *purL, bioF*, and *sucA* mutants all contained wild type copies of their chromosomes in addition to chromosomes where the gene was inactivated by the transposon. Such mutants may easily revert to the wild type, and any analyses of growth or alginate production would be hampered by perceivable fluctuations of the relative copy-number of each chromosome between different cells in the population. Moreover, complementation results of such mutants are not easily interpreted, since any perceived wild-type phenotype theoretically could be caused by a high copy-number of the wild type chromosome rather than by the complementing plasmid.

Still, the phenotypes of the *dxs-1, purL, bioF*, and *sucA* mutants indicate that a lack of essential vitamins or other key metabolites negatively affects *A. vinelandii* alginate production, even when growth is only mildly affected or not affected at all. If this is the case, it is also possible that insufficient access to such compounds is a limiting factor for alginate biosynthesis in the reference strain.

In light of this, we settled on an alternative approach to further assess the impact of these mutations. We hypothesized that sufficient availability of compounds like succinate, biotin, thiamine, or purines could be necessary for alginate biosynthesis, and thus also be a limiting factor for alginate biosynthesis in the reference strain. To investigate this, strain ATCC12518Tc was cultivated in RA1 medium with different supplements: succinate, the four vitamins pyridoxal, pyridoxine, thiamine and biotin, the purine adenine, and a mixture of the three amino acids diaminopimelate, lysine, and methionine (Table 3). The amino acids were chosen due to their dependence on the succinate-derivative succinyl CoA for biosynthesis. While this is true for diaminopimelate and lysine; in *A. vinelandii* methionine biosynthesis is catalyzed by MetX instead of MetA, and probably utilizes acetyl-CoA and not succinyl-CoA as an acyl-donor (Ferla and Patrick, 2014).

**Table 3:** Growth medium combinations used for cultivation experiments to assess nutrient supplements.

**Growth medium**	**Carbon source**	**Additional nutrient supplement(s)**
RA1	Fructose	None
RA1	Fructose	Succinate
RA1	Fructose	Pyridoxine (vitamin B6)
RA1	Fructose	Pyridoxal (vitamin B6)
RA1	Fructose	Thiamine (vitamin B1)
RA1	Fructose	Thiamine and pyridoxine
RA1	Fructose	Thiamine and pyridoxal
RA1	Fructose	Adenine
RA1	Fructose	Lysine, methionine and diaminopimelate
RA1	Fructose	Biotin (vitamin B7)

**Figure 2:**
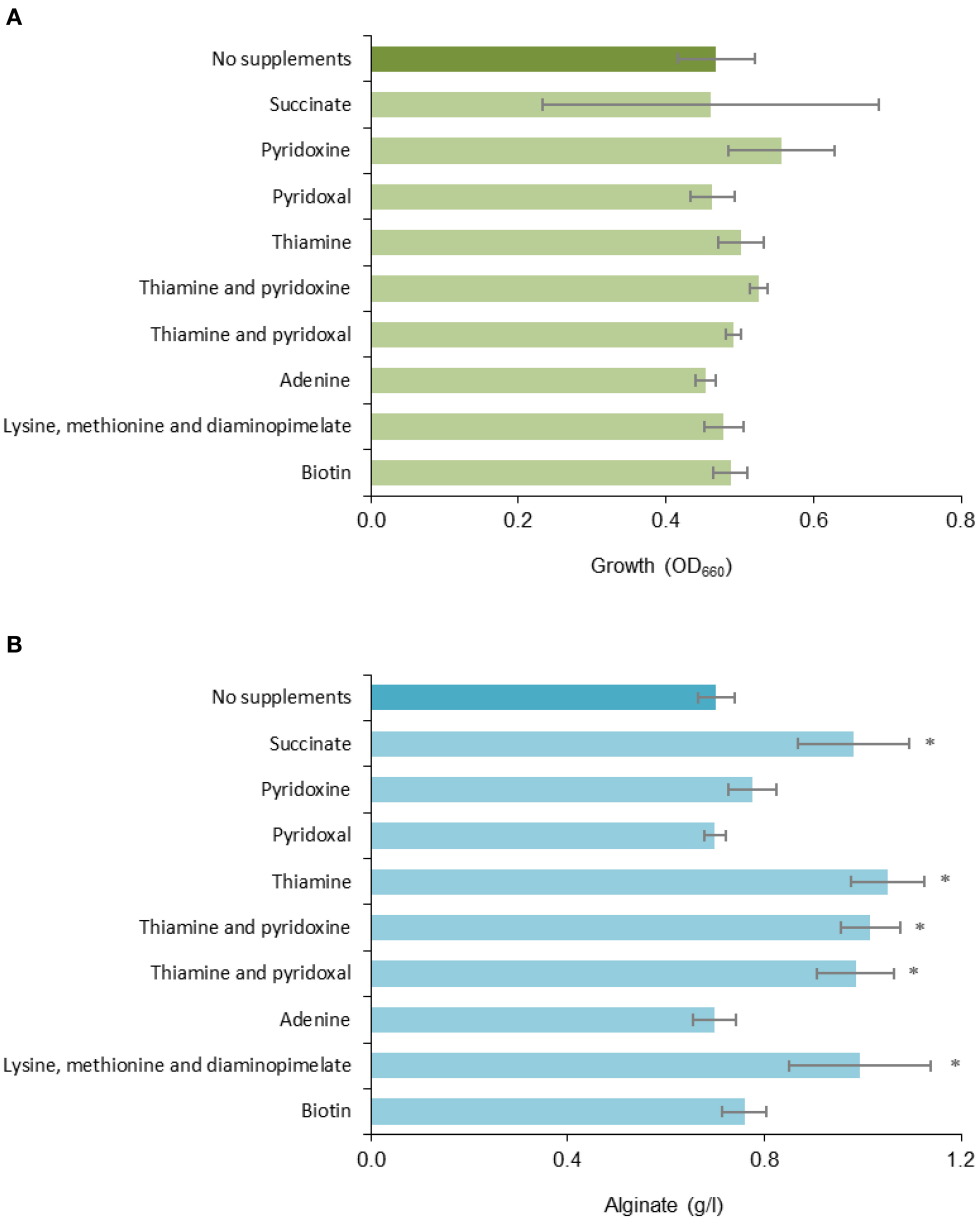
Effect of different media supplements on **(A)** growth and **(B)** alginate production. Triplicate cultures of *A. vinelandii* ATCC12518Tc was grown in deepwell plates containing RA1 with TMS1, fructose and different supplements for 48 h before sampling. Cultures without supplements were included as a reference. Error bars represent one standard deviation. *denotes a statistically significant increase in alginate concentration relative to the reference (*p* ≤ 0.05).

The cultivation results showed that while cell growth was not significantly affected by any of the added nutrients (Figure 2A), addition of succinate, thiamine or a mixture of lysine, methionine, and diaminopimelate increased the amount of measured alginate in the cultures by ~40% (Figure 2B). The observed effect of succinate supplementation could be due to the added carbon, since ~5 g/l was added to the medium (in addition to the main carbon source; 20 g/l fructose), but cell growth did not increase relative to the reference. The effect of the amino acid supplement could also be related to the role of diaminopimelate as a precursor in peptidoglycan biosynthesis (Mengin-Lecreulx et al., 1996), as there appears to be a connection between peptidoglycan metabolism and alginate biosynthesis (Figure 1). Thus, the availability of thiamine and possibly TCA cycle intermediates appears to be limiting for alginate production in *A. vinelandii*.

## Discussion

The presented screen had limited coverage, since several genes known to be necessary for alginate biosynthesis were not identified (confer Figure 1B). Still, genes not previously known to be required for alginate production were identified as necessary, the most interesting of which may be *fruA*. The scarcity of mutants with a reproducible increase in alginate production could reflect the fact that the reference strain, ATCC12518Tc, produces quite large amounts of the polysaccharide, so there might be fewer single gene mutations that will have a pronounced positive effect on production levels. The current study focused on two groups of genes; genes with assumed regulatory functions and genes connected to central metabolism.

### Mutations in Regulatory Genes

Alginate production is dependent on the RpoE-like sigma factor AlgU. MucA is an anti-sigma factor, which sequesters AlgU. A *mucA* mutant was identified in the present study, and as could be expected, this mutant showed an increase in alginate production levels. In *P. aeruginosa*, release of AlgU is controlled by a proteolytic cascade similar to the activation of RpoE in *E. coli*, and AlgW is needed for the first proteolytic cleavage (Delgado et al., 2018). A non-alginate producing *algW* mutant was identified in the present study but could not be complemented by plasmid pHE536. More studies on the role of AlgW and other proteases in *A. vinelandii* are needed in order to elucidate the proteolytic cascade for AlgU activation in this organism.

While the two-component response regulator AlgB is necessary for alginate production in *P. aeruginosa*, our study indicates that this is not the case in *A. vinelandii*. A possible explanation would be that the mutant expresses a truncated AlgB protein which has retained sufficient function to carry out its role in alginate synthesis. This is however not very likely, since the transposon insertion splits the gene almost evenly in half, and a specific amino acid in the C-terminal end has previously been shown to be necessary for the binding of AlgB to P*algD* in *P. aeruginosa* (Ma et al., 1998; Leech et al., 2008). The AlgB proteins from *P. aeruginosa* PAO1 and *A. vinelandii* DJ are of equal length and share 75% sequence identity.

In *P. aeruginosa* the cognate histidine kinase of AlgB is KinB, but AlgB phosphorylation is not needed for its role in alginate production (Ma et al., 1998). KinB has recently been shown to be a negative regulator of alginate biosynthesis in this organism, as AlgW-dependent proteolysis of MucA does not occur in the presence of KinB (Damron et al., 2009). However, no histidine kinase gene is found in the vicinity of *algB* in *A. vinelandii*, and a homology search of the *A. vinelandii* DJ genome revealed that it does not encode a KinB homolog. The phenotype of our mutant and the absence of a KinB homolog indicate that the *algB* gene may not have a role in the regulation of alginate biosynthesis in *A. vinelandii*.

AmrZ was originally identified as a positive regulator for alginate biosynthesis in *P. aeruginosa* (Baynham and Wozniak, 1996). The protein was later found to also act as a positive or negative regulator for several other genes, that encode proteins involved in motility, polysaccharide biosynthesis, iron homeostasis, c-di-GMP production etc., in various species of *Pseudomonas* (See Martínez-Granero et al., 2014; Prada-Ramírez et al., 2016; Hou et al., 2019, and references therein). Interestingly, the effect of AmrZ differs between species; while a *P. aeruginosa amrZ* mutant had an increased level of c-di-GMP (Hou et al., 2019), a *P. fluorescens amrZ* mutant displayed severely reduced levels (Muriel et al., 2018). The effect on motility is also species dependent, from severe defects to hypermotility (Baltrus et al., 2018). The present study shows that *amrZ* is necessary for alginate production in *A. vinelandii*. AmrZ has not previously been studied in *A. vinelandii*, and given the pleiotropic effects of its homolog in *Pseudomonas* spp., it would be interesting to further investigate the role of AmrZ in *A. vinelandii*.

### FruA Is Necessary for Alginate Biosynthesis

Our growth studies showed that FruA is necessary for alginate production on several carbon sources, also those that do not require this protein for uptake. Many carbohydrate PTS systems have regulatory roles besides their function in sugar uptake and phosphorylation (reviewed in Deutscher et al., 2006), which is another possible explanation for the absence of alginate production in this mutant. Similarly to *P. putida* (Velázquez et al., 2007), *A. vinelandii* encodes two PTS systems; FruA-FruB (PTS^Fru^) and PtsO-PtsN-PtsP (PTS^Ntr^). The latter is not involved in carbohydrate uptake, but is believed to be involved in coordinating nitrogen and carbon metabolism in several bacteria and has been shown to affect various metabolic processes (reviewed in Deutscher et al., 2006). Furthermore, cross-talk between the PTS^Fru^ and PTS^Ntr^ systems has been demonstrated in *P. putida* (Pflüger-Grau et al., 2011). In *A. vinelandii*, the PTS^Ntr^ system has been shown to be involved in regulation of polyhydroxybutyrate and alkylresorcinol synthesis (Segura and Espín, 1998; Noguez et al., 2008; Muriel-Millán et al., 2015). As these studies were carried out on derivatives of the *A. vinelandii* UW strain, which does not produce alginate, it is not known whether PTS^Ntr^ mutations also have an effect on alginate synthesis. However, the screening of a *P. fluorescens* transposon insertion library (Figure 1) revealed that inactivation of *ptsP* does affect alginate production negatively in this strain. Thus, our results indicate that the *A. vinelandii* PTS^Fru^ system could have a role in regulation of alginate biosynthesis, possibly via interaction with the PTS^Ntr^ system.

### Alginate Biosynthesis Seems to Be Regulated by the Metabolic Status of the Cell

Our earlier studies in *P. fluorescens* indicated that even in a *mucA* mutant, alginate biosynthesis does not start immediately after the first alginate biosynthetic complexes have been assembled (Maleki et al., 2016). Moreover, both the *P. fluorescens* transposon insertion study (Ertesvåg et al., 2017) and studies focusing on the synthesis of fructose 6-phosphate (Maleki et al., 2015, 2017) clearly indicated that the cells prioritize growth and survival over alginate production. The present study indicates that the same is true for *A. vinelandii*.

Some of the mutants identified in our screen had insertions in genes encoding proteins homologous to or in the same metabolic pathways as proteins identified and confirmed as relevant for alginate biosynthesis in the *P. fluorescens* screen (Figure 1). Even if many of these *A. vinelandii* mutants were not complemented, the concurrence makes it more plausible that the observed phenotypes are due to the introduced mutations.

*A. vinelandii Avin08040* and an *mpl* homolog appear to form an operon, with *Avin08040* located upstream of *mpl*. The transposon insertion in *Avin08040* resulted in decreased alginate production, and a slight decrease has also been observed for a *P. fluorescens mpl* mutant (Ertesvåg et al., 2017). The murein peptide ligase Mpl is involved in the recycling of cell wall peptidoglycan (Mengin-Lecreulx et al., 1996), a process where degradation products from peptidoglycan can be used to resynthesize more peptidoglycan or be utilized as an energy source (Park and Uehara, 2008). *Avin08040* encodes a probable aromatic acid decarboxylase of unknown function, but the observed phenotype of this mutant could result from polar effects on *mpl* expression; a defect in peptidoglycan recycling would impose a drain on fructose-6-phosphate, making less available for alginate biosynthesis. It is also possible that Avin08040 plays a hitherto unknown role in utilization of peptidoglycan degradation products.

Results obtained in the current study show that *dxs-1* is necessary for alginate biosynthesis in *A. vinelandii*. Negative impact on alginate production has also been reported for a *P. fluorescens ispA* mutant (Ertesvåg et al., 2017). For *P. fluorescens* this could be due to a polar effect on *dxs* expression, as these genes are part of the *xseB-ispA-dxs* gene cluster encoding exodeoxyribonuclease VII small subunit, geranyl transferase (isoprenoid biosynthesis), and 1-deoxy-D-xylulose-5-phosphate synthase (thiamine, pyridoxine, and isoprenoid biosynthesis). *P. fluorescens* has one such cluster (Winsor et al., 2016) while the *A. vinelandii* genome contains two nearly identical copies (Setubal et al., 2009). The two *A. vinelandii* Dxs proteins are identical except for one Pro to Ala substitution, but according to our results the bacterium does not produce alginate when only *dxs-2* is intact. Alginate production was restored by complementation with wt *dxs-1*, but not by addition of pyridoxine, pyridoxal or thiamine to the growth medium. This suggests that the role of Dxs-1 in alginate biosynthesis could be related to its role in isoprenoid biosynthesis, or to some yet unknown function of this protein.

We were unable to isolate pure mutants with insertions in the *sucA, purl*, and *bioF* genes. Still, in light of the results from *P. fluorescens*, the phenotype of the *A. vinelandii purL* mutant was as expected and demonstrates that *A. vinelandii* mutants can exhibit changed alginate phenotypes even if the mutant strain is not genetically pure. Based on the expected deficiencies in the identified metabolic mutants, cultivation of the parent strain was performed with addition of selected medium supplements (Table 3). The results (Figure 2) show that adding thiamine, succinate or a mixture of lysine, methionine, and diaminopimelate resulted in increased alginate biosynthesis for the wild type strain. There is no obvious connection between thiamine or succinate levels and alginate biosynthesis, but both compounds are essential for several central metabolic processes. A deficiency can thus be expected to cause suboptimal performance of the cellular metabolism, which could render the cell unable to carry out certain energy-demanding secondary processes, such as biosynthesis of exopolysaccharides.

Furthermore, it was recently shown that *A. vinelandii* cells cultivated using succinate as the sole carbon source produce more alginate and also show differential expression of sRNA genes belonging to the GacS/A-Rsm regulatory system (affects *algD* expression) compared to cells cultivated with glucose or fructose (López-Pliego et al., 2018). Interestingly, succinate, methionine, diaminopimelate and lysine have been shown to lower the thiamine requirement in *Salmonella typhimurium*, probably by reducing the need for succinyl-CoA synthase (Enos-Berlage and Downs, 1997). Since thiamine biosynthesis is competing with purine biosynthesis for precursors, this is an indirect link to alginate biosynthesis (Figure 1). Succinyl CoA is also a precursor for protein succinylation, and such posttranslational modification is used to fine-tune the metabolism (Yang et al., 2015). In addition to proteins involved in TCA and gluconeogenesis, four proteins necessary for alginate biosynthesis, namely AlgU, MucB, AlgR, and AlgC, were found to be succinylated in *P. aeruginosa* (Gaviard et al., 2018).

## Conclusions

Screening of the described *A. vinelandii* transposon mutant library identified only one strain, a *mucA*::TnCAM140 mutant, which produces significantly more alginate per cell than the reference strain. Several gene disruptions resulting in decreased or abolished alginate production were however identified, including genes not previously known to affect alginate biosynthesis. The data also provided new insights regarding alginate regulatory genes in *A. vinelandii* and the cellular processes and metabolites that influence alginate synthesis in this organism. Finally, analyses of the data resulted in the identification of nutrients that limit alginate production in the reference strain, which would not have been easily found using metabolic modeling. The results obtained in the present study will be valuable both for development of alginate bioproduction media and for genetic engineering of *A. vinelandii* toward optimization of alginate production and yield.

## Data Availability Statement

All relevant data is contained within the manuscript.

## Author Contributions

MM, HS, TE, SV, and HE participated in the design of the study. MM, ØJ, HS, GK, and AT performed the experiments. MM and HE reviewed bioinformatic information and literature on relevant genes and drafted the manuscript. All authors read and approved the manuscript.

### Conflict of Interest

The authors ØJ, HS, GK, AT, and TE were employed by the Research Foundation SINTEF Industry. All authors declare that the research was conducted in the absence of any commercial or financial relationships that could be construed as a potential conflict of interest.

## Supplementary Material

Supplementary Figure S1 Gene context of the genes discussed in the Paper: The discussed genes are shown as blue arrows, neighboring genes as red arrows, and the transposon insertions by bars. The genes are sorted by their mutation identifier.
